# The Emerging Roles of E3 Ligases and DUBs in Neurodegenerative Diseases

**DOI:** 10.1007/s12035-022-03063-3

**Published:** 2022-10-19

**Authors:** Na Liu, Miao-Miao Lin, Yan Wang

**Affiliations:** Department of Pharmacology College of Pharmaceutical Sciences, Suzhou Key Laboratory of Aging and Nervous Diseases, and Jiangsu Key Laboratory of Neuropsychiatric Diseases, Soochow University, Suzhou, Jiangsu China

**Keywords:** Neurodegenerative diseases, Ubiquitination, E3 ubiquitin ligases, Deubiquitinating enzymes

## Abstract

Despite annual increases in the incidence and prevalence of neurodegenerative diseases, there is a lack of effective treatment strategies. An increasing number of E3 ubiquitin ligases (E3s) and deubiquitinating enzymes (DUBs) have been observed to participate in the pathogenesis mechanisms of neurodegenerative diseases, on the basis of which we conducted a systematic literature review of the studies. This review will help to explore promising therapeutic targets from highly dynamic ubiquitination modification processes.

## Introduction

The increasing prevalence of neurodegenerative diseases is partly due to increasing human lifespans. However, there is currently a lack of definitive treatment or cure. Although some drugs, surgery, and multidisciplinary treatment can relieve symptoms, their pathogenesis and therapeutic targets require further research [1, 2].

Ubiquitination is the covalent attachment of ubiquitin (Ub) to lysine residues of the substrate [3]. Initially, most scientists believed lysosomes were the only mechanism for degrading proteins that did not require energy. By the late 1970s and early 1980s, scientists Hershko, Ciechanover, and Rose discovered that a polypeptide called ubiquitin plays an important role in energy-dependent protein degradation. They further discovered the mechanism of the ubiquitin-dependent protein-degrading system and were awarded the Nobel Prize [4–6]. As an enzymatic, posttranslational modification, ubiquitination is involved in several critical biological processes, such as proteasomal degradation of proteins, signal transduction, the cell cycle, transcriptional regulation, and DNA repair [7, 8]. Ubiquitination occurs through a three-step sequential enzymatic cascade mediated by E1 ubiquitin-activating enzymes (E1s), E2 ubiquitin-conjugating enzymes (E2s), and E3 ubiquitin ligases(E3s). Deubiquitinating enzymes (DUBs) catalyze Ub removal from targeted substrates [7, 9]. Complicated and diverse topologies of ubiquitin chains provide a structural basis for the transmission of biological signals. Additionally, ubiquitin can bind to different sites and be modified by other posttranslational modification processes, such as acetylation and phosphorylation [10]. Specific E3 ubiquitin ligases (E3s) and DUBs play key regulatory roles as “pens” and “erasers,” respectively. They regulate the structure and properties of ubiquitin chains, forming complex and diverse signal transduction systems that tightly regulate important processes in living organisms [11].

In the aging process, the balance between ubiquitination and deubiquitination strictly regulates neuronal homeostasis and has a profound impact on neuronal survival. A hallmark of various neurodegenerative diseases is an abnormal accumulation of neurotoxic proteins, such as α-synuclein in Parkinson’s disease (PD), amyloid-β (Aβ) and tau in Alzheimer’s disease (AD), and mutant huntingtin (mHTT) in Huntington’s disease (HD) [12–15]. E3s and DUBs are closely linked to the clearance and degradation of proteins, such as proteasomal degradation, autophagy, and endoplasmic reticulum (ER) phagocytosis, which suggests that their dysfunction contributes to the pathology of neurodegenerative diseases [11, 16]. Furthermore, E3s and DUBs convey complex and unique biological signals that regulate cellular processes associated with the pathology of neurodegenerative diseases, such as mitochondrial function, excitotoxicity, and immune inflammation [11, 17]. The roles of E3s and DUBs in neurodegenerative diseases have been extensively investigated (Tables 1 and 2; direct interactors/substrates of E3s/DUBs; for each disease, the list is sorted alphabetically by E3s/DUBs).

**Table 1:** E3s in neurodegenerative diseases

Disease	E3 ligase	Interactor/substrate	Functional implication of the E3 ligase	Reference
PD	CHIP	α-synuclein	Promote the degradation of α-synuclein by UPS or lysosomes	[18]
PD	CHIP	G2385R LRRK2	Promote G2385R LRRK2 degradation	[19]
PD	Nedd4	α-synuclein	Promote α-synuclein degradation	[20]
PD	Parkin	NEMO	Upregulate mitochondrial OPA1 transcription to maintain mitochondrial integrity	[21, 22]
PD	Parkin	RIPK1	Activate NF-κB and MAPKs	[23]
PD	Parkin	Multiple targets	Induce mitophagy	[24]
AD	CHIP	BACE1	Reduce APP processing	[25]
AD		Tau	Promote tau proteins degradation	[26]
AD	CRL	BRI2/3	Inhibit APP processing and Aβ oligomerization	[27]
AD	Itch	Tap73	Regulate cell cycle	[28]
AD	Mdm2	Cav1.2	Regulate calcium homeostasis	[29]
AD	PIAS1	Endogenous APP	Reduce Aβ and amyloid deposition	[30]
HD	CHIP	mHTT	Promote mHTT degradation	[31]
HD	FBXW7	HSF1	Promote HSF1 degradation	[32]
HD	HACE1	NRF2	Increase antioxidant capacity	[33]
HD	HOIP	mHTT	Promote mHTT degradation	[34]
HD	PIAS1	HTT	Regulate PNKP activity	[35]
HD	UBE3A	mHTT	Promote the degradation of mHTT by UPS	[36]
ALS	CHIP	Mutant SOD1	Promote mutant SOD1 degradation	[37, 38]
ALS	GP78	Mutant SOD1	Promote mutant SOD1 degradation	[39]
ALS	SYVN1	OPTN	Promote the degradation of OPTN by UPS	[40]

Only the direct interactors/substrates for E3s and DUBS are listed. For each disease, the list is sorted alphabetically by “E3 ligase”

**Table 2:** DUBs in neurodegenerative diseases

Disease	DUB	Interactor/substrate	Functional implications of the DUB	Reference
PD	USP8	α-synuclein	Prevent lysosomal degradation of α-synuclein	[41]
PD	USP10	P62	Induce synaptic aggregates formation	[42]
PD	USP24	ULK1	Downregulate autophagic flux	[43]
PD	USP33	Parkin	Inhibit mitophagy	[44]
AD	OTUB1	Tau	Promote tau protein stability and aggregation	[45]
AD	USP46	AMPARs	Regulate synaptic receptor levels	[46]
HD	ATXN3	Beclin-1	Regulate autophagy	[47]
ALS	USP7	Nedd4L	Regulate SMAD-mediated protein quality control system	[48]

Only the direct interactors/substrates for E3s and DUBS are listed. For each disease, the list is sorted alphabetically by “DUB”

**Fig. 1:**
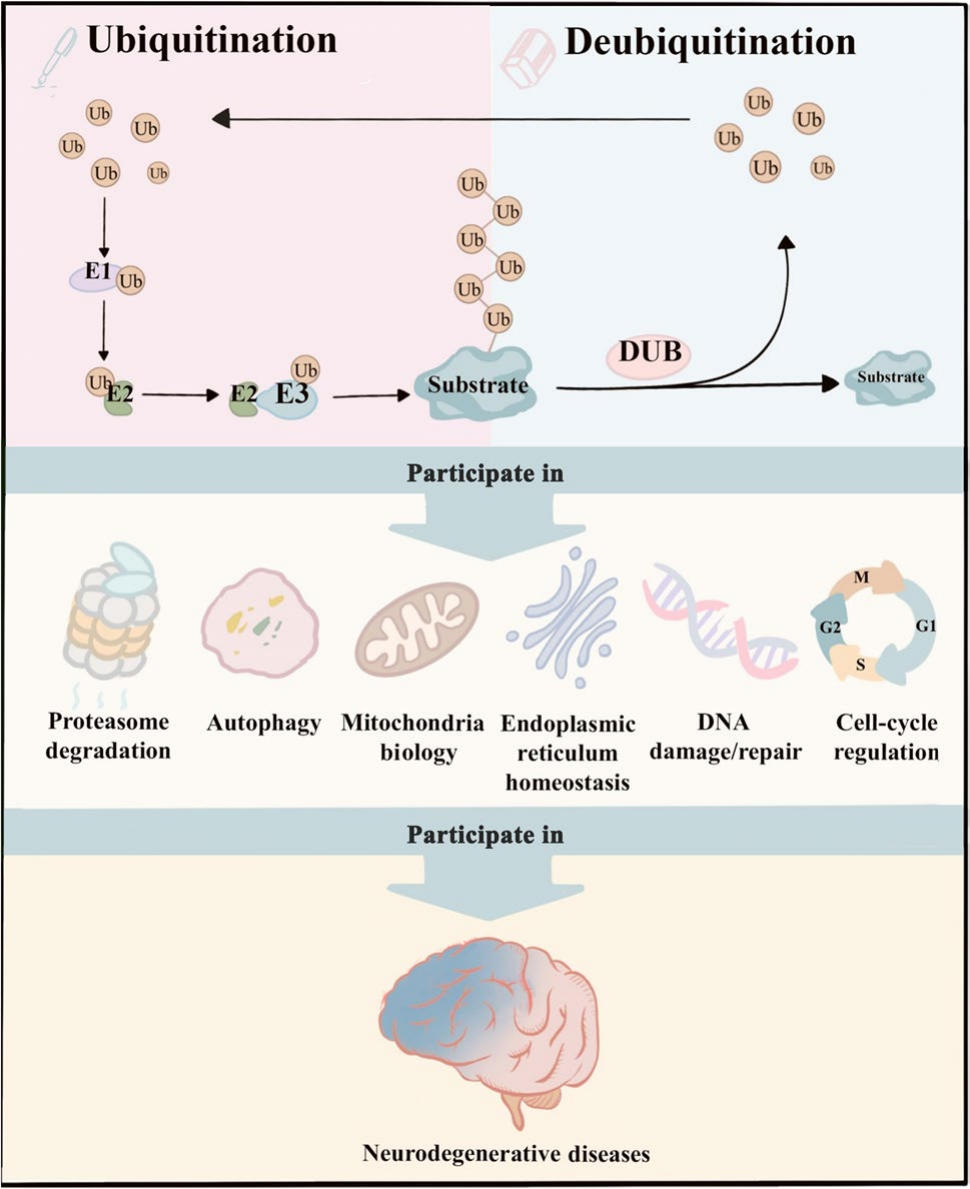
E3 ubiquitin ligases and DUBs act as “pens” and “erasers” to write the ubiquitin code and are involved in the regulation of protein homeostasis, autophagy, mitochondrial biology, endoplasmic reticulum homeostasis, DNA repair, cell cycle regulation, and other physiological processes. They are closely associated with the occurrence and development of neurodegenerative diseases. DUB, deubiquitinating enzyme; E1, E1 ubiquitin-activating enzyme; E2, E2 ubiquitin-conjugating enzyme; E3, E3 ubiquitin ligase; Ub, ubiquitin. This schematic is created with BioRender.com

In this review, we summarize the emerging roles of E3s and DUBs in neurodegenerative diseases and elaborate on the pathogenesis from the perspective of ubiquitin signaling regulation to identify promising therapeutic targets (Fig. 1).

## E3s Involved in Neurodegenerative Diseases

Three enzymes play vital roles in the ubiquitination machinery. A high-energy thiol ester bond is formed between the cysteine residue of E1 and the glycine residue of ubiquitin, which is powered by ATP. The activated ubiquitin is then transferred to the cysteine residue of E2. E3 catalyzes the final step of the ubiquitin cascade by transferring E2-bound ubiquitin to substrates. It catalyzes the isopeptide bond between the glycine residue of ubiquitin and the lysine residue in the substrate. The cascade is repeated until a complete ubiquitin chain is assembled [7, 9].

E3s are the most critical and heterogeneous enzymes involved in the process of ubiquitination; they specifically recognize target proteins. Bioinformatics analysis has revealed that the human genome encodes more than 600 E3s [49]. Based on different characteristic domains and ubiquitin transfer mechanisms, there are primarily three types of E3s, namely, the really interesting new gene (RING), homologous to the E6AP C-terminus (HECT), and the RING-between-RING (RBR) E3s [49]. Recent studies indicate that U-box-containing proteins form another class of E3s. The U-box structure is similar to the RING domain except that it lacks zinc-binding mods and requires a salt bridge to become stable [50].

### E3s in PD

PD was first described as “shaking palsy” in 1817 by James Parkinson, a British physician [51]. It is the second most common neurodegenerative disease characterized by motor symptoms, including bradykinesia, muscular rigidity, gait impairment, postural impairment, and rest tremor. In addition to motor symptoms, non-motor features also commonly accompany PD. Non-motor features include psychiatric symptoms, olfactory dysfunction, sleep disorders, cognitive impairment, autonomic dysfunction, fatigue, and pain [52].

In the early twentieth century, spherical eosinophils named Lewy bodies were detected in patients’ substantia nigra neurons and were the first pathologic feature of PD to be identified [53]. In the late 1950s, Carlsson discovered that dopamine is an important neurotransmitter in brain tissue and that its deficiency contributes to the occurrence and development of PD [54]. PD is currently characterized by dopamine neuronal death in the substantia nigra pars compacta and amyloid-like aggregate formation of α-synuclein, also known as Lewy bodies [55, 56]. Mutations in leucine-rich repeat kinase 2 (BACCdk), parkin (PARK2), and PTEN-induced kinase 1 (PINK1) are the most frequently known etiologies of familial PD [57, 58]. There is extensive evidence documenting the correlation between E3- and DUB-regulated ubiquitination modification and pathogenic mutations in PD.

#### E3s Linked to α-Synuclein in PD

The carboxy-terminus of Hsc70-interacting protein (CHIP) is a dimeric U-box E3 ligase that is widely expressed in the central nervous system. It is closely related to the pathogenesis of neurodegenerative diseases [26]. CHIP degrades α-synuclein directly by targeting the ubiquitin-proteasome system (UPS) through the tetrapeptide repeat domain and mediates lysosomal phagocytosis through the U-box domain. It differentially influences abnormal protein aggregates through the protein quality control function of Hsp70/Hsp90-based chaperone machinery. The amino-terminal TPR domain of CHIP binds to either Hsp70 or Hsp90 and exerts opposite effects on target proteins. Hsp70 promotes CHIP-mediated UPS activity, whereas Hsp90 plays the opposite role by inhibiting ubiquitination and stabilizing the substrate [18].

Neural precursor cell expressed, developmentally downregulated 4 (Nedd4) belongs to the NEDD-type HECT ligase family. NRBPIt has been observed that Nedd4 ubiquitinates α-synuclein directly and promotes homeostasis of endosomal transport in Lewy bodies in brain samples collected from PD patients [20]. N-aryl benzimidazole (NAB2), a compound targeting Nedd4, can effectively reduce the aggregation and toxicity of α-synuclein [59].

#### E3s Linked to Mitochondrial Function in PD

Some studies suggest that deficiency in mitophagy and mitochondrial dynamics contributes to the pathology of PD [24, 60–62]. Mitophagy is a type of selective autophagy that depends on polyubiquitination modification. Parkin is an RBR E3s and acts as a key regulator of mitophagy. In depolarized mitochondria, accumulated PINK1 on the mitochondrial outer membrane phosphorylates ubiquitin to stimulate parkin and recruit it to mitochondria [63, 64]. Parkin assembles ubiquitin chains (Lys 6, Lys 11, and Lys 63) on the outer membrane of damaged mitochondria and mediates mitochondrial sequestration through interaction with adaptor proteins on the separation membrane [64–66]. Several mitochondrial-mapped DUBs constantly function against this process by deconstructing ubiquitin chains from mitochondria until parkin’s ubiquitin activity takes over. For example, ubiquitin-specific protease 30 (USP30) prefers removing K6- and K11-linked polyubiquitin chains [65, 66].

In addition to *parkin*, mutations in *LRRK2* are another common genetic cause of familial PD [57]. Under ER stress, LRRK2 regulates the activities of E3 ubiquitin ligases in a PERK kinase activity-dependent manner, including membrane-associated ring finger (C3HC4) 5 (MARCH5), MUtability LANdscape inference (MULAN), and parkin. MARCH5, MULAN, and parkin localize to mitochondria and ubiquitinate mitochondria-associated membrane (MAM) components to regulate MAM formation and mitochondrial genesis [19]. CHIP ubiquitinates different regions of LRRK2, thereby mediating its degradation. *G2385R LRRK2* has a higher affinity for CHIP than wild-type *LRRK2* [19]. These findings suggest that CHIP is an ideal candidate target for PD treatment.

Multiple familial Parkinson’s disease–related gene studies conducted on several populations have identified mutations in *F-box domain-containing protein (Fbxo7)* [67], which is the substrate recognition component of the Skp1-Cullin-F-box protein E3 ubiquitin ligase complex [68]. Studies on Fbxo7 in PD have primarily focused on the maintenance of mitochondrial function. Stress upregulates endogenous Fbxo7 expression, which in turn induces Fbxo7 aggregates in mitochondria and impairs mitochondrial bioenergetics. High expression and aggregation of Fbxo7 have been found in brains from PD or AD patients. However, *Fbxo7* defects induce NAD^+^ deficiency and poly (ADP-ribose) polymerase (PARP) overactivation, eventually resulting in impaired mitochondrial respiration and mitochondrial dysfunction [68]. In addition, recent studies found reduced proteasome activity and early‐onset motor deficits together with premature death in *Fbxo7* knockout mice [69]. The role of Fbxo7 expression in mitochondria and PD requires further exploration to reach consensus.

#### E3s Linked to Neuroinflammation in PD

In PD, there is ongoing and end-stage neuroinflammation, as demonstrated by neurohistological and neuroimaging studies. In addition, changes in inflammatory markers and immune cell populations may initiate or exacerbate neuroinflammation and perpetuate neurodegenerative processes [70]. Mitochondrial stress in *PINK1*- and *parkin*-knockout mice leads to STING-mediated type I interferon responses, which supports a role for PINK1/parkin-mediated mitophagy in restraining innate immunity [61]. Recruitment of parkin into mitochondria also increases linear ubiquitination of nuclear factor-kB (NF-kB) essential modulator (NEMO), which then upregulates transcription of mitochondrial guanosine triphosphatase *optic atrophy 1* (*OPA1*) to maintain mitochondrial integrity. This mechanism links NF-kB to mitochondrial integrity through linear ubiquitination [21]. In addition, parkin also modulates the K63 ubiquitination status of RIPK1 to promote the activation of NF-κB and mitogen-activated protein kinases (MAPKs) [23].

#### E3s Linked to Excitotoxicity in PD

Excitotoxicity is prevalent in various neurodegenerative diseases [71]. Activity of the Nedd4-2 ubiquitin ligase mediates abnormal transport of glutamate translocator induced by 1-methyl-4-phenyl-1,2,3,6-tetrahydropyridine (MPTP) and upregulates excitotoxicity. Knockdown of *Nedd4-2* was found to improve motor dysfunction and glial proliferation in MPTP-treated mice [72].

#### E3s Linked to ER Stress in PD

Mutation of *parkin* is a genetic cause of PD. Pael receptor (Pael-R) is a substrate of parkin and leads to ER stress when it accumulates in the ER of dopaminergic neurons. CHIP enhances the dissociation of Hsp70 from parkin and Pael-R, accelerating Pael-R ubiquitination mediated by parkin. Overexpression of CHIP enhances parkin-mediated ubiquitin degradation of Pael-R, which inhibits neuronal death induced by ER stress [26].

#### E3s Linked to Apoptosis in PD

X-linked IAP (XIAP) is the most widely expressed IAP and has three BIR domains and one ring domain. The BIR domain possesses anti-caspase activity, and the ring domain mediates the E3 ubiquitin ligase activity of XIAP [73]. The RING domain of XIAP can be S-nitrosodized by nitric oxide. S-nitrosylated XIAP has been detected in the brains of patients with various neurodegenerative diseases. In PD patients and animals, increased nitrosylation impairs the anti-apoptotic ability of XIAP, but does not affect its E3 ubiquitin ligase activity [74]. However, another study claimed that S-nitrosylation of XIAP downregulates its own E3 ligase activity, thereby negatively regulating the anti-apoptotic function of XIAP [75]. Its role as an E3 ligase in PD remains unclear.

### E3 Ligases in AD

Alzheimer’s disease (AD) is a progressive neurodegenerative disease and the most common form of dementia. The clinical features of the patient are initial memory impairment and cognitive decline, and later impairment of behavior, visuospatial orientation, and motor system [76]. Extracellular Aβ plaques and intracellular neurofibrillary tangles containing tau proteins were discovered in the brains of AD patients in 1984 and 1986 respectively, and they were established as the pathological diagnostic criteria for AD [77, 78].

#### E3s Linked to Aβ and Tau in AD

CHIP is essential for amyloid-β precursor protein (APP)-induced autophagy dysfunction. Depletion of *CHIP* effectively alleviates pathological symptoms induced by APP in flies. β-site app-cleaving enzyme 1 (BACE1) catalyzes the rate-limiting step of Aβ generation and is considered to be a prime target for AD [79]. Nevertheless, CHIP overexpression downregulates BACE1 by promoting ubiquitin-dependent degradation, thus reducing APP processing. This process is dependent on the U-box and TPR domains of CHIP [26]. This means that CHIP is two-sided, both inhibiting APP clipping and contributing to APP-induced autophagy dysfunction [25]. In AD, *CHIP* overexpression directly ubiquitinates and degrades tau proteins and reduces their phosphorylation. CHIP is more susceptible to conjugate tau^D421^ proteins than to full-length tau. CHIP inhibits caspase-6 ubiquitin independently, which contributes to the homeostasis of tau. Interestingly, a study demonstrated that CHIP mediates the effects of Aβ on tau proteins. Aβ42 downregulates CHIP expression and hinders tau protein degradation, which can be alleviated by restoring CHIP expression. These studies confirm that CHIP mutations play an essential role in AD pathology [26].

Homodimeric nuclear receptor binding protein 1 (NRBP1) assembles into Cul2- and Cul4A-containing heterodimeric Cullin-RING ubiquitin ligase (CRL). NRBP1-containing CRL2/CRL4A targets integral membrane protein 2b (ITM2b, also known as BRI2) and brain protein I 3 (BRI3) for degradation, which inhibits APP processing and Aβ oligomerization [27]. BRI2 depends on its extracellular Brichos domain to reduce Aβ aggregation and tau phosphorylation in AD. A *BRI2* mutant designed with a stable monomer state effectively protected against Aβ42-induced neurotoxicity [80]. In AD, both BRI2 and BRI3 reduce Aβ and amyloid deposition in lesions. However, the effects exerted by BRI2 and BRI3 are inconsistent. The efficiency of the Brichos domain of BRI3 in reducing Aβ production and neurofibrillary tangle formation is significantly lower than that of the domain of BRI2 [81, 82].

Protein inhibitor of activated STAT 1 (PIAS1) ubiquitinates the endogenous APP intracellular domain to enhance its combination with Fe65 and nuclear translocation, leading to a reduction in Aβ and amyloid deposition, as well as the activation of neprilysin and transthyretin, two major Aβ-degrading enzymes, respectively [30]. PIAS1 also mediates SUMOylation of the ETS transcription factor ELK1, an endogenous defense regulator against Aβ in APP/PS1 mice [83]. RNA sequencing was conducted to evaluate and quantify the gene expression profiles in response to *PIAS1* overexpression in HT-22 cells. Five transcription factor–binding site genes that were significantly downregulated were identified, including early growth response 1 (Egr1), a downstream target of NF-kB in neurons. In this study, the authors constructed a regulatory network for *PIAS1* overexpression, including nuclear receptor subfamily 3 group C member 2 (NR3C2), which directly interacts with PIAS1. However, further studies on these downstream targets are necessary [84].

Recent studies have demonstrated that E3 ubiquitin ligase activity is related to the phagocytosis of Aβ aggregates through microglial cells in AD. Pellino E3 ubiquitin protein ligase 1 (Peli1) is upregulated in the microglia of *5* × *FAD* mice and directly ubiquitinates CCAAT/enhancer-binding protein β (C/EBPβ) to inhibit its function in blocking CD36 transcription. Consequently, Aβ phagocytosis by microglia is decreased [85].

#### E3s Linked to Mitochondrial Function in AD

*PARK2* mutations can be detected in patients with tauopathies [86, 87]. In transgenic mice with overexpression of mutant human *FTDP-17 Tau* and knockout of *PARK2*, parkin deficiency results in abnormal hyperphosphorylated tau protein aggregates [88]. Impaired mitochondrial clearance occurs early in AD progression, and several studies have examined the regulation of mitophagy by parkin in AD [89–91]. Pathological tau proteins disrupt mitochondrial physiology, including mitochondrial quality control, by inhibiting parkin recruitment into defective mitochondria. Tau pathology and mitochondrial disorders promote each other, forming a vicious cycle [87].

#### E3s Linked to Calcium Overload in AD

Cav1.2 plays a vital role in calcium overload and neuronal death in AD [29, 92]. In *APP/PS1* double-mutant mice, the E3 ligase MDM2 proto-oncogene (Mdm2) facilitates Cav1.2 ubiquitination and degradation in vivo, and ultimately improves cognitive function. The expression of Mdm2 can be upregulated by estrogen receptor α (ERα) agonists [29]. Another Nedd4 family member, Nedd4-1, is required by Aβ to reduce the density and synaptic strength of AMPA receptors on the plasma membrane [93, 94].

#### E3s Linked to the Cell Cycle in AD

Anaphase-promoting complex/cyclosome (APC/C) is a multisubunit E3 ubiquitin ligase that regulates cell cycle by assembling multiubiquitin chains onto regulatory proteins and degrading them. Cyclin B1, which is the substrate of the E3 ligase APC/C, has been found to accumulate in brain lesions of AD patients. In cortical neurons with NMDA receptors overstimulation, cyclin-dependent kinase-5 (Cdk5) mediates the phosphorylation of Cdh1 and inactivates downstream APC/C, ultimately resulting in the accumulation of cyclin B1 in the nucleus. Cyclin B1 accumulation induced apoptosis in neurotoxicity [95]. Aβ treatment induces increased levels of supernatant glutamate in primary neurons. In the absence of the APC/C-Cdh1 complex, neurons tend to suffer excitotoxicity induced by glutamate. Pharmacological inhibition of glutaminase, a known target of ubiquitin ligase, reverses this process [96].

In AD mice and Aβ42-treated neurons, the Nedd4 family E3 ligase Itch exerts a regulatory role in the apoptosis of terminally differentiated neurons induced by the abnormal cell cycle [28]. In Aβ42-treated neurons or neurons from an AD transgenic mouse model, secondary to the activation of the JNK pathway, Itch hyperphosphorylation induces its own ubiquitination, thereby promoting TAp73 degradation. TAp73 participates in the transcription of genes that inhibit cell cycle progression and negatively regulate neuronal apoptosis caused by cell cycle re-entry [28].

### E3 Ligases in HD

HD is an inherited neurodegenerative disorder characterized by movement disorders (most common dance form), neuropsychiatric symptoms, and progressive cognitive impairment [97]. The pathological hallmark of HD is an expansion mutation of trinucleotide *CAG* in exon 1 of the *huntingtin* gene (*HTT*) [98]. The gene is localized to human chromosome 4 by genetic linkage [99]. Mutant *HTT* (*mHTT*) is abnormally modified after translation, resulting in disturbed transcription and immune and mitochondrial functions. Mutant HTT is the earliest biomarker that can be detected in the serum of patients with HD [15].

#### E3s Linked to mHTT in HD

In astrocytes, increased activity of monoubiquitinated CHIP promotes the K48-linked polyubiquitination and degradation of mHTT. However, in neurons, CHIP activity is inhibited by high expression of HSPA (Hsp70)-binding protein 1 (HspBP1). This may be the reason why neurons are more sensitive to external stress than astrocytes in HD and other neurodegenerative diseases [31].

The Skp1-Cul1-F-box (SCF) complex is one of the most typical ubiquitin ligases and plays a role in maintaining the integrity of postmitotic neurons. SCF deficiency contributes to the pathology of polyglutathione (polyQ) diseases. In *R6/2* transgenic mouse models of HD, Cul1 and Skp1 are downregulated. Overexpression of *Cul1* exerts a dominant negative effect on mutant huntingtin aggregation [100].

Ubiquitin protein ligase E3 component n-recognin 5 (UBR5) is a HECT domain E3 ligase. Studies conducted on *Caenorhabditis elegans* and human cell lines have demonstrated that UBR5 promotes proteasomal amplification of normal and polyQ-amplified HTT, depending on its ubiquitination activity. Silent *UBR5* results in mHTT aggregates in HD iPSCs. Other E3s highly expressed in iPSCs have not been reported to exert such a role, such as UBR7, ubiquitin protein ligase E3A (UBE3A), and RNF181. They deserve attention and study [101].

Heat shock transcription factor 1 (HSF1) expression plays a vital role in the clearance of mHTT aggregates and is significantly reduced in brains from HD patients. In HD mouse models, mHTT upregulates F-box and WD repeat domain-containing 7 (FBXW7), resulting in the degradation of HSF1 via ubiquitin-dependent degradation. Phosphorylation of S303 and S307 by CK2α′ kinase is required for the interaction of HSF1 with Fbxw7, which is responsible for ubiquitin-dependent degradation of HSF1 in pathogenic polyQ-expressing cells and tissues [32].

PIAS1 is believed to selectively regulate the accumulation of mHTT and sumoylated proteins. PIAS1 specificity enhances HTT modification by SUMO-1 and SUMO-2, leading to increased insoluble HTT aggregation. *PIAS1* deficiency is found to significantly improve the behavioral phenotype and microglial activation in the R6/2 HD mouse model [102, 103]. PIAS1 also interacts with HTT to regulate polynucleotide kinase–phosphatase (PNKP) activity and genomic stability in vivo. *PIAS1* deletion upregulates PNKP activity, an important protein for DNA damage repair (DDR) in HD. The PIAS1-DDR pathway is important for the progression of HD [35].

UBE3A is decreased in aged mouse brain. Aging-dependent UBE3A levels result in differential ubiquitination and degradation of HTT fragments, thereby contributing to the age-related neurotoxicity of mHTT. In both in vitro and in vivo HD models, the E3 ligase UBE3A degrades mHTT by K63-mediated ubiquitination and targets it to the UPS [36].

#### E3s Linked to Antioxidants in HD

In striatal tissue from HD patients, HECT domain and ankyrin repeat–containing E3 ubiquitin protein ligase 1 (HACE1) expression levels are downregulated. A study demonstrated that HACE1 can activate nuclear factor erythroid 2 like 2 (NRF2) and increase its antioxidant capacity by promoting NRF2 protein synthesis, stabilization, and nuclear localization. This requires HACE1 ankyrin repeats as well as its HECT domain, but is independent of its E3 ubiquitin ligase activity [33].

In the polyubiquitin chain, the C-terminal glycine of donor ubiquitin is generally connected to one of the seven lysine residues of receptor ubiquitin by an isopeptide bond or to the N-terminal methionine of receptor ubiquitin by a peptide bond, causing linear or M1 ubiquitination [104]. This type of ubiquitin ligation is generated only by a ubiquitin E3 ligase complex known as the linear ubiquitin chain assembly complex (LUBAC), which is to date the only E3 ligase capable of forming linear ubiquitin chains. LUBAC consists of two RBR E3s, HOIP and RanBP-type and C3HC4-type zinc finger containing 1 (Rbck1, also known as HOIL-1 L) [105]. HOIP is the catalytic active component of LUBAC and the only known E3 ubiquitin enzyme that can assemble linear ubiquitin chains because of a unique ubiquitin-binding domain between its C-terminal and RBR domains [106]. In HD, linear ubiquitin chains are enriched in HTT aggregates. HOIP is collected into misfolded HTT aggregate through its N-terminal PUB domain in a P97/VCP-dependent manner, thus promoting the effective recruitment of chain quality control components. HOIP-catalyzed linear ubiquitination can enhance the clearance of HTT-polyQ and reduce protein toxicity [34].

### E3 Ligases in ALS

ALS is an adult-onset motor neuron disorder that is characterized by progressive motor symptoms, such as muscle weakness, muscle atrophy, and spasticity [107]. The dominant mutant *superoxide dismutase 1* (*SOD1*) is the first gene identified to cause ALS. Misfolded SOD1 forms ubiquitinated cytoplasmic inclusions that accumulate as the disease progresses. In parallel, another mechanism is the aggregation of TAR DNA-binding protein (TARDBP, also known as TDP43) in the cytoplasm due to mutations in *TARDBP* and repeated amplification of *C9orf72* [108].

#### E3s Linked to Mutant SOD1 in ALS

*SOD1* mutation is one of the common mutational causes of familial ALS [109]. CHIP selectively and indirectly promotes the degradation of mutant *SOD1*, but it has no significant impact on wild-type SOD1, which is dependent on the HSP-mediated chaperone mechanism [37, 38].

Ring finger protein 19A (Rnf19a, also known as dorfin) is the first identified E3 ligase that can specifically ubiquitinate SOD [26]. Previous results indicated that dorfin ubiquitylates mutant SOD1 and improves disease phenotypes. However, there has been limited new research since then [37].

GP78 is a RING E3 that can promote proteasome-dependent degradation of mutant SOD1 proteins and is involved in ER-related degradation. GP78 expression is upregulated in cells transfected with mutant SOD1 as well as in ALS mice [39]. Overexpression of *GP78* promotes ubiquitination and degradation of SOD1 and protects cells against mutant SOD1 and ataxin-3-induced ER stress and neurotoxicity [39].

#### E3s Linked to TDP43 in ALS

*CCNF* mutations exist in patients with sporadic ALS. CCNF encodes cyclin F, a component of an E3 ubiquitin ligase SCF^cyclin F^ complex that is responsible for ubiquitylating proteins for degradation by the UPS. Mutant cyclin F disrupts ubiquitylation at Lys48, resulting in the accumulation of substrates and autophagic defects that are implicated in ALS pathogenesis. Mutant cyclin F has also been reported to cause abnormal ubiquitination and accumulation of TDP43 [110, 111].

#### E3s Linked to Mutant OPTN in ALS

Synoviolin 1 (SYVN1) promotes ubiquitin–proteasome-dependent degradation of misfolded proteins in the ER-associated process [40]. Mutations in *OPTN* are associated with ALS. In two variants of *OPTN*, E50K *OPTN* is found to be more unstable than the other variant, E478G *OPTN*. SYVN1 induces ubiquitination-dependent degradation of wild-type and E50K OPTN. Interestingly, when UPS is blocked, SYVN1 may instead promote the aggregation of wild-type and E478G OPTN [112]. The exact underlying mechanism remains unclear.

## DUBs Involved in Neurodegenerative Diseases

DUBs can specifically remove ubiquitin from substrates to reverse the ubiquitination process. Monomeric modifiers produced by DUBs process ribosome fusion and polyubiquitin cassettes to generate free ubiquitin. The former is a mechanism whereby resting cells maintain ubiquitin, and the latter can rapidly release ubiquitin under stress. Free ubiquitin is then captured by ubiquitination machinery to maintain the operation of the ubiquitination system [7].

In mammals, approximately 100 DUBs depolymerize and remove ubiquitin adducts. DUBs are divided into six families according to the conserved sequence and domain as follows: ubiquitin-specific proteases (USPs); ubiquitin carboxy-terminal hydrolases (UCHs); Machado–Josephin domain-containing proteases (MJDs); ovarian tumor proteases (OTUs); motif-interacting with ubiquitin-containing novel DUB family (MINDYs); and JAB1, MPN, MOV34 family (JAMMs). DUBs are cysteine peptidases, except for JAMM, which is a zinc metallopeptidase [113].

### DUBs in PD

#### DUBs Linked to α-Synuclein in PD

Mass spectrometry studies have demonstrated the presence of OTU deubiquitinase, ubiquitin aldehyde binding 1 (OTUB1) in Lewy bodies of PD patients, and in amyloid plaques of AD patients [45, 114]. OTUB1 has several intrinsic properties of amyloid, forming inclusion bodies in neurons during rotenone-induced cytotoxicity. This oligomer destroys the neuronal membrane and cytoskeleton and simultaneously upregulates the expression of α-synuclein. This suggests that OUTB1 is cytotoxic and contributes to PD pathologically by forming α-synuclein [45].

YOD1 deubiquitinase (YOD1) is an important deubiquitination enzyme involved in ER stress–induced degradation [115]. It has been found that YOD1 is upregulated by both mutant mHTT and aggregated α-synuclein. The location of YOD1 has been observed in the Lewy bodies of patients with PD [116]. YOD1 inhibits Lewy body formation and its toxicity through deubiquitination activity [117].

In DA neurons, ubiquitin-specific protease 8 (USP8) is located in the Lewy body and uncouples the K63-linked ubiquitin chain on α-synuclein. It prevents lysosomal degradation of the aggregating proteins and increases their toxicity, which may be the key mechanism underlying α-synuclein accumulation [41]. Therefore, USP8 may contribute to the occurrence and development of PD pathology.

Ubiquitin regulates the distribution of α-synuclein in different protein degradation systems. Monoubiquitination preferentially mediates proteasome-dependent degradation, whereas deubiquitination more likely induces autophagy. In general, when monoubiquitination is dominant, the α-synuclein level is downregulated, and when deubiquitination prevails, the event goes the other way, suggesting that UPS is the primary pathway for the degradation of α-synuclein [118]. In PD, ubiquitin-specific protease 9X (USP9X)-mediated deubiquitination regulates the degradation of α-synuclein. In the substantia nigra of PD patients, both the expression and deubiquitinase activity of USP9X are found to be significantly reduced, and α-synuclein tends to be allocated to the proteasome compartment. Impaired proteasome function in PD causes aggregative monoubiquitination of α-synuclein. In this case, enhanced autophagy coupled with activation of USP9X deubiquitinase activity may alleviate symptoms by enhancing degradation [118, 119].

Previous research has demonstrated that ubiquitin-specific protease 10 (USP10) interacts with P62 in PD to induce the formation of synaptic aggregates, including α-synuclein [42]. Overlocalization of USP10 with toxic protein aggregates has been found in the brains of both PD and AD patients. This finding suggests that USP10 is a key factor in regulating the production of harmful aggregates and their toxic effects in neurodegenerative diseases [120].

*Ubiquitin-specific protease 13 (USP13)* overexpression can be observed in PD patient brains. USP13 knockdown increases α-synuclein ubiquitination and leads to clearance of α-synuclein-containing vacuoles via the lysosome [121]. In animal models and cell culture, USP13 independently regulates the E3 ubiquitin ligase parkin, which is associated with autosomal recessive PD. Knocking down *USP13* increases ubiquitinated α-synuclein and promotes its clearance independent of parkin [122].

#### DUBs Linked to Mitochondrial Function in PD

Ubiquitin C-terminal hydrolase L1 (UCH-L1) is the first DUB found to possess neuronal function. In AD and PD, UCH-L1 is modified by oxidation, resulting in reduced solubility, low hydrolytic activity, and increased accumulation [123, 124]. Several studies have investigated the relationship between UCH-L1 and PD. In vitro and in vivo, parkin mediates K63-linked polyubiquitination of UCH-L1, increasing its degradation through the autophagy–lysosomal pathway. The PD-associated *parkin* mutation weakens this linkage [125]. It has been demonstrated that *UCH-L1* knockdown can downregulate Mfn2 in different cell lines, causing mitochondrial enlargement and tubular network collapse, as well as reduced mitochondria-ER connection and Ca^2+^ absorption. This effect depends on the cytoplasmic localization and deubiquitination activity of UCH-L1. As PD is highly associated with mitochondrial biology and quality control, UCH-L1 may be a target for regulating mitochondrial function in PD [126].

Ubiquitin-specific protease 14 (USP14) maintains free monomer ubiquitin storage and regulates autophagy and proteasome activity in the nervous system. Targeting USP14 has demonstrated some efficacy in neurodegenerative diseases [127]. Inhibitors of USP14 are found to reduce cerebral ischemia/reperfusion-induced neuronal damage and improve motor function recovery by reducing protein aggregation and enhancing proteasome activity [128]. Joy Chakraborty first reported that the specific inhibitor IU1 and *USP14* knockout protected against mitochondrial dysfunction in *PINK1/parkin*-deficient models. It enhanced mitophagy by promoting a PINK1/parkin-independent pathway to clear damaged mitochondria, emphasizing its potential in PD treatment [129]. The potential of USP14 inhibitors in PD rodents and key factors to determine the administration regimen have been reported [130].

Previous research has reported that ubiquitin-specific protease 15 (USP15) antagonizes parkin-mediated mitochondrial ubiquitination and that *USP15* knockout can rescue mitophagy impairment in PD patient brains with *PARK2* mutation [131].

USP30 is a key factor involved in the ubiquitin system to regulate mitochondrial import and mitophagy [132–134]. In oxygen–glucose deprivation/reperfusion (OGDR) models, *USP30* overexpression inhibits OGDR-induced ubiquitination and degradation of Mfn2 and reduces mitochondrial fragmentation [135]. Mitochondrial dysfunction plays a vital role in the pathological process of PD. In dopaminergic neurons, *USP30* overexpression reduces rotenone -induced cell death, although it does not improve dopamine-treated cells [136].

Ubiquitin-specific protease 33 (USP33) is another direct target of parkin and antagonizes parkin’s role as a precursor for phagocytosis. USP33 deubiquitinates Lys435 of parkin and inhibits its mediated clearance of damaged mitochondria. Knocking out *USP33* reduces the degradation of parkin, increases its recruitment to depolarized mitochondria and upregulates mitophagy. USP33 inhibitors may be candidates for regulating mitochondrial function in PD [44].

#### DUBs Linked to Neuroinflammation in PD

However, studies have demonstrated that USP8 promotes the transformation of microglia from the M1 phenotype to the M2 phenotype through the TLR4/MyD88/NF-KB pathway, thereby alleviating inflammation and movement disorders induced by LPS [137]. Neuroinflammation is one of the typical pathological features of PD [138]. Therefore, the effect of USP8 in PD must be further investigated.

#### DUBs Linked to Excitotoxicity in PD

Excitotoxicity is a common event in various neurodegenerative diseases and involves calcium overload, oxidative stress, mitochondrial damage, and other pathological processes [139, 140]. Recent studies have demonstrated that inhibiting USP15 prevents glutamate-induced oxidative stress and neuronal apoptosis by activating the NRF2/heme oxygenase 1 (HO-1) signaling pathway in HT22 cells [141].

#### DUBs Linked to Autophagy in PD

A meta-analysis identified novel susceptibility genes for PD to validate the previously nominated candidate genes within the PARK10 region, of which ubiquitin-specific protease 24 (USP24) is one [142]. The association between single-nucleotide polymorphisms in USP24 and PD was also verified in a Han population [132]. A quantitative high-throughput screening in the human genome-wide siRNA library identified USP24 as one of the candidate genes regulating autophagy. USP24 expression is upregulated in the substantia nigra of PD patients, suggesting that USP24 exerts its negative effect on autophagy in PD [141]. USP24 deubiquitinates ULK1, an autophagy mediator downstream of mTOR, inhibits its activity and downregulates autophagic flux. This conclusion has been confirmed in IPSC-derived human dopaminergic neurons [43]. However, the effect of USP24 on autophagy in a specific animal or cell model of PD remains to be investigated.

### DUBs in AD

#### DUBs Linked to Aβ and Tau in AD

Neurofibrillary tangles (NFTs) and neural plaques are characteristic of AD. Polypeptides in NFT total homogenates were identified by tandem mass spectrometry. The results indicate that UCH-L1 colocalizes with highly phosphorylated tau proteins [143]. Aβ42 activates the NF-kB pathway and downregulates UCH-L1. This process blocks the brain-derived neurotrophic factor (BDNF)/neurotrophic receptor tyrosine kinase 2 (NTRK2)-mediated retrograding signal, reduces the degradation of ubiquitination and hyperphosphorylated tau proteins, and promotes inflammation [144].

Genome-wide association studies have identified OUTB1 as a deubiquitinase at Lys48 of tau proteins that upregulates their phosphorylation and oligomerization. It increases tau protein stability and aggregation. Inhibitors targeting OUTB1 may provide novel therapies for AD [114].

A study demonstrated that knocking down *USP8* can upregulate BACE1 ubiquitination-mediated lysosomal degradation in H4 cells and reduce the production of Aβ, which provides an idea for AD treatment [145].

As mentioned earlier, USP10 is excessively colocalized with toxic proteins in the brains of AD patients [120]. USP10 has been demonstrated to be a key factor in the formation of tau-positive stress granules in neurons. In HT22 cells, *USP10* overexpression induces TIA1/Tau/USP10-positive SGs. It is important to note that this process is independent of deubiquitinase activity [146].

In transgenic animal models overexpressing murine tau proteins, *USP13* knockdown reduces amyloid levels and increases *p*-tau ubiquitination, allowing its clearance by autophagy/proteasome [147].

#### DUBs Linked to Excitotoxicity in AD

Excitotoxicity is a common event in neurodegenerative diseases and plays a vital role in the occurrence and development of diseases [148]. Reversible ubiquitination of AMPARs regulates synaptic receptor levels and synaptic strength. In *C. elegans* and mammals, the WD40-repeat protein WDR-20 binds to WDR-48 and activates USP46. Ubiquitin-specific protease 46 (USP46) deubiquitinates the glutamate receptors GLR-1 and AMPAR, resulting in increased surface levels of these receptors [149, 150]. Downregulation of AMPAR expression is one of the early pathologies of AD. In AD brains and neurons incubated with Aβ, USP46 expression is downregulated, triggering ubiquitination, and clearance of AMPARs. It has been suggested that USP46 dysfunction is one of the reasons for the downregulation of AMPARs in AD [46]. However, in HD, intervention with USP46 does not rescue mHTT-mediated neurodegeneration [151]

### DUBs in HD

#### DUBs Linked to mHTT in HD

Ataxin-3 (ATXN3) is a DUB closely related to protein quality control and is important for ataxia type 3 and other polyQ diseases. PolyQ-amplified ATXN3 continues to bind and cleave the polyQ chain. In addition to maintaining protein homeostasis, ATXN3 is involved in regulating autophagy, DNA damage and repair, microglial activation, and other pathologies associated with polyQ disorders [152]. Together with ATXN3, DNA repair enzyme polynucleotide-kinase-3′-phosphatases (PNKP) and cyclic AMP-response element-binding protein (CBP), HTT causes transcription-coupled DNA repair (TCR), which can identify damage in the template DNA chain and mediate its repair in the process of transcriptional extension [153]. PolyQ amplification in mHTT impairs the activities of sATXN3 and destroys the functional integrity of the TCR complex, thus being detrimental to transcription and DNA repair [154]. Moreover, low ATXN3 activity increases CBP ubiquitination and degradation, which negatively influences CREB-dependent transcription [154]. Therefore, therapies targeting ATXN3 may be effective against polyQ diseases, including HD. To ensure normal autophagy, the polyQ domain of wild-type ATXN3 enables it to interact with Beclin-1 and protects Beclin-1 from proteasome-mediated degradation, depending on ATXN3’s deubiquitination enzyme activity. Other soluble proteins with polyQ fragments competitively bind to Beclin-1 with wild-type ATXN3, e.g., full-length huntingtin protein amplified by mutant polyQ; this results in impaired autophagy in mHTT-expressing cells or animal models [47].

Abnormal interactions between soluble mHTT oligomers encoded by the mutated exon 1 fragment and other proteins have been analyzed, wherein ubiquitin-specific protease 7 (USP7) is one of the subjects. Western blotting analysis of striatal and cortical lysates from mice indicated that USP7 interacts with both wild-type and mutant HTT but preferentially with polyQ-amplified HTT [155]. However, PLA analysis of patient-derived cells showed no significant differences, possibly because the heterozygosity of the HTT allele masks differences in this interaction. PolyQ expansion within the androgen receptor (AR) causes progressive neuromuscular toxicity in the spinal cord and medullary muscular atrophy (SBMA). An analysis of the interacting genomes indicated that USP7 preferentially interacts with polyQ-amplified AR in vitro and in vivo, especially soluble aggregates. Knocking out *USP7* rescues polyQ amplification-induced AR aggregation and improves other characteristic pathologies of SBMA in *Drosophila*, depending on its deubiquitinase activity [155]. The critical role of USP7 in the pathophysiology of SBMA suggests a similar role in HD. Further experiments are required to clarify the underlying mechanisms and the significance of differences in USP7 interactions with wild-type and mutated HTT proteins.

Ubiquitin-specific protease 12 (USP12) has a specific inhibitory effect on mHTT toxicity, rescuing mHTT-mediated neurodegeneration in an animal or *Drosophila* model of HD. This effect cannot be replaced by USP46, nor can it be reproduced in the neurotoxicity induced by TDP43 and α-synuclein. USP12 inhibits mHTT neurotoxicity independent of its deubiquitinase activity. This suggests that USP12 has a unique noncatalytic function in addition to deubiquitination [151]. Recent studies have demonstrated that USP12 plays a neuroprotective role by inducing autophagy in HD models. This may be because polyQ-containing proteins interfere with Beclin-1-induced autophagy, and USP12 specifically compensates for the mHTT-related defects in autophagy [151]. The conserved association between USP12 and mHTT is worthy of further investigation aimed at revealing the specific mechanism [151].

An imbalance of ubiquitin levels may partly contribute to HD pathology [156]. In the above-described context, downregulating USP14 expression is preferable in treating PD and AD. In cells and animals expressing mHTT, *USP14* overexpression inhibits the phosphorylation-dependent activation of IRE1α, which is a serine-threonine kinase involved in ER stress and reduces insoluble mHTT [157]. The effects of USP14 on different protein aggregates are quite different, and this warrants further exploration of the reasons for this difference [157].

As mentioned earlier, linear ubiquitination helps reduce the toxicity of the mHTT protein. OTULIN is a specific deubiquitinating enzyme required for linear polyubiquitination. Silencing OTULIN significantly reduces HTT-Q97-induced toxicity by reducing c-Jun phosphorylation and caspase-3 activation [34].

### DUBs in ALS

#### DUBs Linked to Mutant SOD1 and TDP43 in ALS

Nedd4 L is an E3 ligase that targets SMAD [48]. Nedd4 L enhances the polyubiquitination and degradation of mutated SOD1 in the spinal cord of ALS patients [158]. USP7 interacts directly with and deubiquitinates Nedd4 L, regulating the SMAD-mediated protein quality control system and the toxicity of SOD1 and TDP-43 [48]. USP7 also interacts with the AR amplified by polyQ to reduce mutant AR aggregation. *USP7* knockout improves motor dysfunction in transgenic SBMA mice [159].

#### Cylindromatosis (CYLD) in ALS

Familial amyotrophic lateral sclerosis accounts for approximately 10% of ALS cases [160]. To date, more than 50 related genes have been identified, among which the most well-known are *SOD1*, *TDP43*, *FUS RNA-binding protein* (*FUS*), and *C9orf72-SMCR8 complex subunit* (*C9orf72*). In recent years, several novel related genes have been identified, and *CYLD* is one of them [161]. CYLD is a deubiquitination enzyme that specializes in removing K63-linked polyubiquitin chains from several substrates [162]. Based on complete gene sequencing of a large European Australian family, a study demonstrated the segregation of a novel missense variant in *CYLD* (c.2155A > G, p. M719 V) within the linkage region as the genetic cause of disease in this family [163]. Another research team detected that the variant g.50825515A > G causes the substitution of methionine with valine at amino acid position 719 of CYLD. This variant is present within the region of overlap with the chromosome 16q12.1-linked ALS pedigree [164]. ALS caused by this gene mutation is still under further exploration and may be associated with the regulation of autophagy [163].

## Targeting Ubiquitin or Deubiquitin Signaling to Treat Neurodegenerative Disease

The above review concludes that dynamic regulation of the ubiquitin system is the key to removing toxic metabolites from neurons. Strategies targeting ubiquitin signal transduction, including substrate recognition, ubiquitin enzymes, DUBs, and proteasome activity, modulate the clearance of toxic or misfolded proteins. Some of the compounds developed have shown promising potential in alleviating neurodegenerative diseases.

### Proteasomal Activators

Activation of proteasomal enzymes allows neurons to maintain a homeostatic state by reducing their proteotoxic burden. Several small molecule agonists of 20S proteasome activity have been developed and are being investigated for therapeutics for neurodegenerative diseases. For example, dihydroquinazolines stimulate three catalytic sites of the 20S proteasome and the degradation of α-synuclein [165]. Pyrazolones were identified as proteasome activators in 2014. They have shown initial therapeutic potential in ALS cells and animal models, and the mechanism of action depends on the activation of the proteasome [166]. The 26S proteasome targets ubiquitinated protein substrates, whereas the 20S proteasome is limited to disordered protein degradation. Few small molecules have been developed that directly activate the 26S proteasome. Modulation of posttranslational modifications and gene manipulation are commonly used to indirectly enhance 26S proteasome, for example, inhibition of DUBs, modulation of camp-dependent protein kinase A (PKA) and CTMP-dependent protein kinase G, and inhibition of P38 mitogen-activated protein kinase (MAPK).

### Allosteric Modification of the Substrate-binding Region

Allosteric modification of the substrate-binding region of the ubiquitin enzyme with small molecules can regulate substrate specificity and thus regulate protein degradation. Such drugs have been used to treat cancers but have rarely been administered in neurodegenerative diseases, such as arginine- and proline-rich peptides, chloroquine and its derivatives, clioquinol, dicarboxylic acid compound (SCF-I2), and pentanoic acid compound (CC0651) [167–170].

### DUB Inhibitors

DUBs affect various neurodegenerative diseases by regulating substrate ubiquitination and equilibrium substrate abundance by lysosomal or proteasome degradation. The administration of DUB inhibitors can enhance the deubiquitination of mutated polyubiquitin chains, thus reducing the substrate burden of the proteasome and facilitating the UPS machinery to function efficiently. DUB inhibitors also protect E1, E2, and E3 enzymes from self-ubiquitination and degradation, ensuring their clearance of harmful proteins under pathological conditions [171].

DUB inhibitors are being progressively developed and studied, and their efficacy in neurodegenerative disease has been partly investigated (Table 3, for each disease, the list is sorted alphabetically by compounds). More data are needed to elucidate the potential regulatory mechanisms of different types of DUB inhibitors in neurodegenerative diseases. To date, the efficacy of DUB inhibitors against other diseases, such as cancer, has also been proven [172]. In contrast, DUB inhibitors have not been adequately studied for neurodegenerative diseases.

**Table 3:** Potential inhibitors of DUBs involved in therapeutic implications in neurodegenerative diseases

Disease	Compounds	Target protein	Functional implication of the compound	References
PD	CMPD-39	USP30	Enhance peroxisome turnover and mitophagy	[173]
PD	FT3967385	USP30	Accelerate PINK1 generation of phospho-Ser65 ubiquitin	[174]
AD	IU1	USP14	Accelerate tau degradation	[175–177]
AD	LDN57444	UCHL1	Inhibit tau aggregation and affect APP processing	[178–180]
ALS	HBX41108	USP7	Inhibit the toxicity of the mutant SOD1 in neurons	[181]

For each disease, the list is sorted alphabetically by “Compounds”

### Development of Proteolysis-targeting Chimeras (PROTAC)

Because of structural similarities between (de)ubiquitinases, the development of substrate-specific drugs still faces challenges. PROTAC, a newly developed technology, is revolutionizing therapies for various neurodegenerative diseases. The PROTAC is composed of hetero bifunctional molecules, including a ligand for an intracellular target protein, a recruiting group for a E3 ligase, and a linker that connects these ligands. PROTACs stimulate the formation of ternary complexes between target proteins and specific E3 ligase. Polyubiquitin modification and 26S proteasome–mediated degradation were subsequently performed. In this process, only target proteins are degraded, while PROTACs do not disintegrate and continue to operate [182, 183]. PROTACs have the advantage of high blood–brain barrier permeability and can be administered in multiple routes. Their potential for treating neurodegenerative diseases such as AD and PD is being explored [184, 185].

Despite the tremendous advances in PROTAC, development challenges remain, such as the lack of ligands for E3 ligase. Currently, only about 1% of human E3s have small molecule ligands. The development of novel ligands will facilitate the wider application of PROTACs to target various pathogenic proteins in neurodegenerative diseases. In addition, the lack of pharmacokinetic data, ternary crystal structure analysis techniques, and reliable bioactivity assessment are also urgent challenges [186].

## Conclusion

Ubiquitination and deubiquitination generally regulate protein levels through ubiquitin-mediated proteasome degradation and are also associated with autophagy, mitochondrial function, apoptosis, and other signal transduction pathways. Over the past few decades, there has been a significant amount of research on the association of E3s and DUBs with neurodegenerative diseases. Numerous studies have disclosed that adjusting their stability or activity through gene mutation or posttranslational modification can cause or inhibit multiple neurological diseases. Several studies have also identified critical E3s and DUBs in PD, AD, HD, and ALS, as well as sites for ubiquitination and deubiquitination of target proteins. However, the underlying mechanisms of some of them remain unclear, and even contradictory conclusions exist. Moreover, a few researchers have considered E3s and DUBs together, which have mutually repelling effects. Further studies are required to gain a complete understanding of the importance of the balance between E3-mediated ubiquitination and DUB-mediated deubiquitination for the development of therapeutic strategies for treating neurodegenerative diseases.

## Data Availability

The authors declare the availability of data and material.
